# Chemical diversity of reagents that modify RNA 2′-OH in water: a review

**DOI:** 10.1039/d4sc05317f

**Published:** 2024-09-12

**Authors:** Ryuta Shioi, Eric T. Kool

**Affiliations:** a Department of Chemistry, Stanford University Stanford CA 94305 USA kool@stanford.edu; b Department of Chemistry, Stanford University Stanford CA 94305 USA

## Abstract

Electrophilic water-soluble compounds have proven versatile in reacting selectively with 2′-OH groups in RNA, enabling structure mapping, probing, caging, labeling, crosslinking, and conjugation of RNAs *in vitro* and in living cells. While early work focused on one or two types of reagents with limited properties, recent studies have greatly diversified the structure, properties, and applications of these reagents. Here we review the scope of documented RNA hydroxyl-reactive species reported to date, with an eye to the effects of chemical structure on reactivity with RNA and other useful properties. Multiple forms of carbonyl electrophiles are now known to react at the 2′-OH, and recently, sulfonyl and aryl electrophiles have also been documented to form bonds there in high yields as well. In addition to electrophilicity, data also point to significant effects of reagent stability, steric bulk, and chirality on reaction yields and selectivity. Finally, we outline reagent properties and principles that define utility in applications with RNA, with an eye to the design of future reagents.

## Abstract 2

Electrophilic water-soluble compounds have proven versatile in reacting selectively with 2′-OH groups in RNA, enabling structure mapping, probing, caging, labeling, crosslinking, and conjugation of RNAs *in vitro* and in living cells.

## Graphical abstract

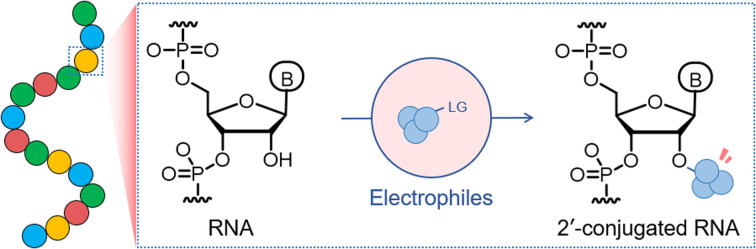

## Introduction

The covalent reaction of small molecules with biomacromolecules is critically important both in basic and applied science. In the protein and proteomics fields, the development of reactions of small activated species with protein sidechains has revolutionized biology and biomedicine. It has made possible the fluorescence labelling of antibodies for biomedical imaging and quantitation,^1,2^ and has enabled the construction of medically important antibody-drug conjugates.^3^ Moreover, the use of simple chemical species reacting with protein sidechains throughout the cell has enabled the characterisation of interactions within the entire proteome.^4,5^

The cellular RNA population, like the protein population, is complex and dynamic, and undergoes a multitude of biologically important interactions in the cell.^5^ We argue that RNA science and applications will likely benefit at least as much as protein studies do from similar bond-forming reactions with small molecules. Trace reactions that are structure-sensitive can be used to profile RNA folded structures and complexes in the cell.^6–8^ Higher-yielding reactions can be used to construct conjugates for labelling, separation, and enrichment applications.^9–16^ They can also be used to control folding and biological activity,^13^ and to stabilise the polymer against degradation.^17^ Finally, covalent reactions with RNA can be employed to profile the interactions of small molecules (such as drugs and other small ligands) with individual RNAs or throughout the whole transcriptome, by providing a covalent trap of local binding interactions.^18–20^

**Fig. 1:**
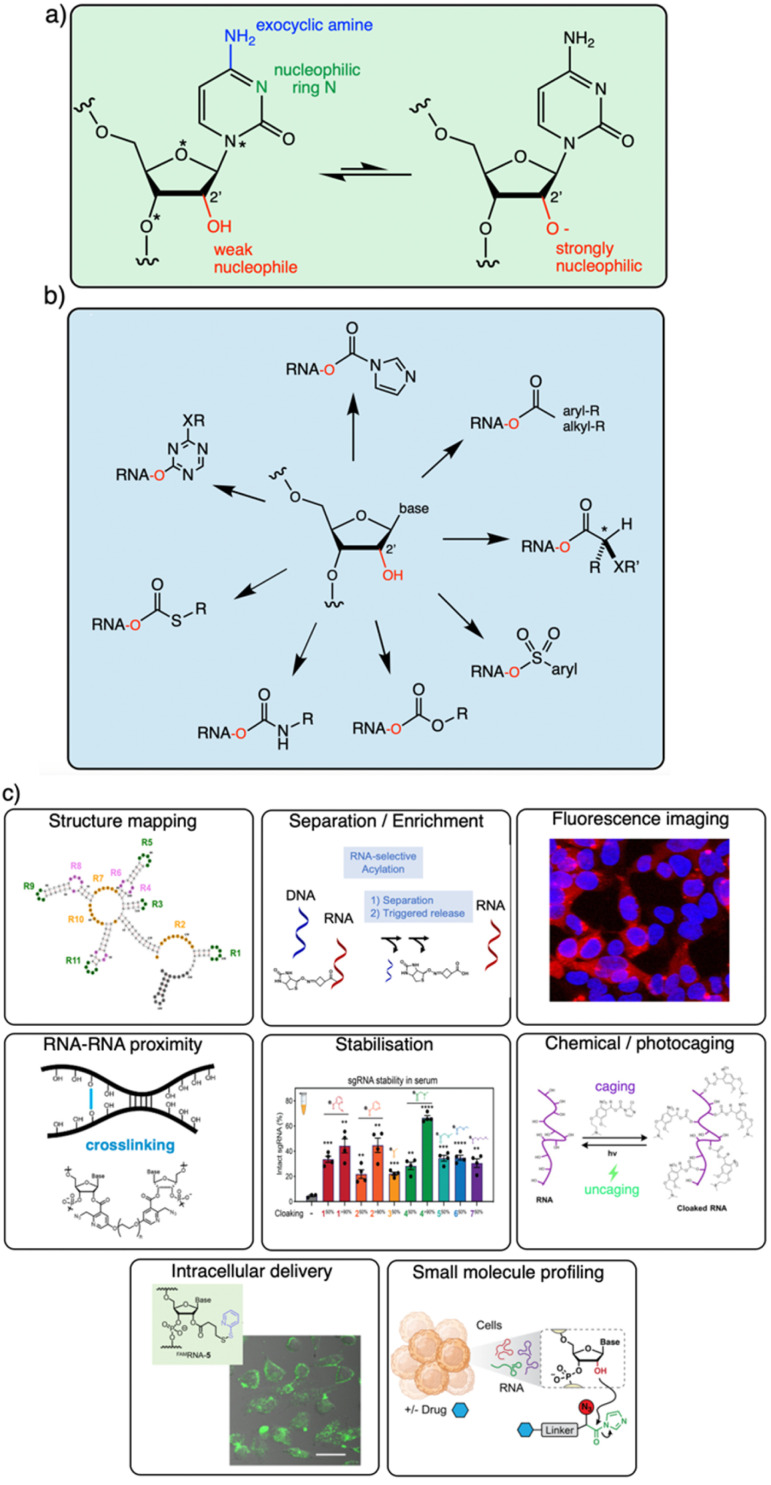
Diverse reactivity of the 2′-OH group in RNA. (a) Structure of cytidine nucleotide in RNA, showing commonly nucleophilic atoms, including N-3 ring nitrogen, exocyclic 4-amine group on the base, and 2′-OH group on the ribose sugar. This review focuses on reagents that are selective for the latter nucleophile on all four nucleotides. Adenosine, guanosine and uridine nucleotides also have similar (although not identical) 2′-OH reactivity. Proximal electronegative atoms (*) in the sugar and base inductively increase the acidity of the 2′-OH, which likely reacts with electrophiles in the rare but rapidly equilibrating anionic form (right).^21,22^ (b) Diversity of established 2′-OH group adduct structures achieved to date, ranging from carbonyl esters (both alkyl and aryl), to sulfonyl esters, carbonates, thiocarbonates, carbamates, and aryl ethers. Adducts can be flat or bulky, achiral or chiral, and can have alkyl or aryl substitution along with a variety of functional groups (X, R). (c) Overview of several biological applications of 2′-OH modification of RNA.

It is becoming increasingly clear that the 2′-OH group offers an extraordinarily versatile chemical handle on RNA (Fig. 1). To be sure, other reaction sites exist on this biopolymer; for example, researchers frequently perform aqueous covalent reactions with nucleobases in RNA. Aldehydes such as glyoxal react with exocyclic amines of some of the bases (particularly guanine),^10,23^ and alkylating species (such as dimethylsulfate or nitrogen mustard) react with nucleophilic nitrogens on adenine and cytosine.^24,25^ In addition, diazirines can be used as carbene precursors, leading to reactions with RNA bases (primarily guanine) under UV irradiation.^26,27^ While useful, those reactions are limited to a subpopulation of nucleobases in RNA, and are nonselective between DNA and RNA. In contrast, the 2′-OH group, absent from DNA, occurs at nearly every position of all RNAs in the cell. This provides an immense opportunity to interrogate and modify RNA at every position along the biopolymer. In addition, reagents targeted to the 2′-OH group have been shown to be highly tuneable, and the resulting adducts can be designed to be reversible,^13,28^ leaving no scar behind afterward.

The goal of this brief overview is to summarise the range of organic reagents that have been documented to react selectively with RNA at 2′-OH groups, with a view to structural variations, reactivity, and other relevant properties (Fig. 1). This is meant to outline for the reader the current state of the art in chemistry for RNA modification, and therefore focuses chiefly on high-yield preparatively useful reactions. Biological applications of 2′-OH reactions are rapidly expanding (see Fig. 1c for several) and have been reviewed recently.^29,30^ In addition, because excellent reviews already exist on the use of isatoic anhydride and acylimidazole reagents in trace-yield reactions for RNA structure mapping,^29–31^ we largely bypass that topic here with only brief mentions, and refer the reader to those sources.^32–34^

### Mechanism of reaction

Successful reagent designs for bond-forming reactions often benefit from mechanistic knowledge of the reaction under study. For esterification of sugars, activated acyl species (*e.g.* acyl chlorides and anhydrides) have long been preparatively useful reagents. Such reactions are typically carried out in the presence of weak bases in organic solvents, excluding water that would compete for the reagent, and the reactions are usually selective for less sterically encumbered primary hydroxyls. In contrast, for RNA in water, it is observed that certain aqueous-soluble acyl species can react in high yields at the 2′-OH groups despite the competition with water itself (see below).^7^ It is notable that the reaction can be selective for this secondary hydroxyl over even the primary 5′-OH group of RNA strands, which is the reverse of the selectivity that is usually observed during esterification of typical alcohols.

This begs the question of what makes the 2′-OH group in RNA specially reactive. McGinnis *et al.* investigated variable furanose conformations and possible general base catalysis of acylation by nearby basic groups on nucleobases to explain “hyper-reactive” hydroxyls.^35^ A recent study concluded that inductive effects of the heteroatoms near the 2′-OH group render it much more reactive, likely due to the increased concentration of the anionic form at neutral pH.^21^ The early data support the notion that acylation occurs by trapping the rare but rapidly equilibrating transient anionic form of the 2′-OH group. We suggest that in some respects, RNA 2′-OH is analogous to the hydroxyl of trichloroethanol, as both have three highly electronegative atoms at similar distances from the reacting alcohol. The p*K*_a_ of RNA 2′-OH has been measured to be ∼12.5,^36^ very nearly the same as that of trichloroethanol.^22^ To be sure, the unusual 2′-OH reactivity is more complex than inductive effects alone, however, and local folding of RNA, sugar and chain conformation, electrostatic and steric effects, and solvation all likely play important roles. More work is needed to better understand these effects.

### Isatoic anhydrides

**Fig. 2:**
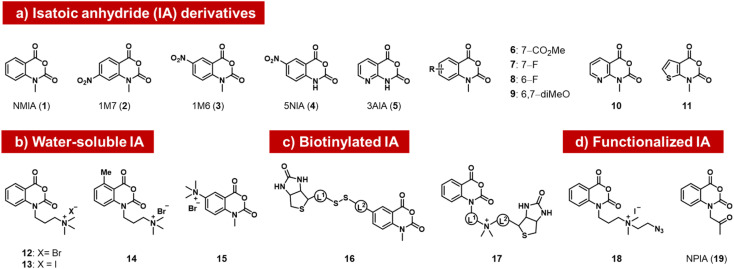
Isatoic anhydrides (IAs) documented as acylating species for RNA, designed and employed for mapping of RNA structure, as well as for labeling RNA with biotin, azide, or ketone groups.

The isatoic anhydrides as a class of ribose-reactive reagents (Fig. 2) were studied early on by Hiratsuka, who documented reactions of mononucleotides with the simple isatoic anhydrides NAIM (1) and 1M7 (2).^37,38^ Weeks made the important observation that reactions of these reagents also could be performed with RNAs in trace yields selectively at single-stranded regions of the polymer over duplex regions, and subsequently developed these reagents into powerful tools for analysis of folded structure, probing local sites of reaction by primer extension with a reverse transcriptase enzyme, which tends to stop at sites of acyl adducts.^39^ The general methodology (selective hydroxyl acylation analysed by primer extension, SHAPE) is widely used for RNA structure mapping, and has been reviewed elsewhere.^34,40^

Subsequently, researchers tested structural variants of the isatoic anhydrides with an aim to expanding their properties. Isatoic anhydrides in these studies are typically prepared in two steps from 2-aminobenzoic acids, using triphosgene to form the anhydride, followed by an *N*-alkylation step to add substituents.^41^ Early reagents 1–5 can be used to generate trace-level reactions for RNA mapping, but were not documented to react in high yields.^42–44^ Possible limiting factors are low aqueous solubility and short half-life in water, which rapidly diminishes concentrations. Half-lives of isatoic anhydrides typically range from a few seconds to 14 minutes depending on functional groups and substitution patterns (6–11).^45^

Modified derivatives, such as biotinylated isatoic anhydrides (*e.g.*16) have also been reported and were employed to separate RNA from DNA in mixtures of the two, demonstrating higher yields of at least one adduct per strand.^14,45^ More recent studies by Fessler and coworkers have reported isatoic anhydrides with appended solubilising groups (*e.g*. 12–15),^46,47^ including azide-functionalised reagent 18, and this has resulted in reactions with favourable preparative-level reactions with RNA (although numeric yields were not reported). Analogous isatoic anhydride reagents with a cationic water-soluble linker and biotin were developed for quantification of protein and modification of RNA (16, 17).^48^ Finally, a ketone-linked isatoic anhydride reagent (19; NPIA) and its further conjugation with a hydrazide have been employed in a novel mapping method for RNA structure.^49^

### Acylimidazoles

**Fig. 3:**
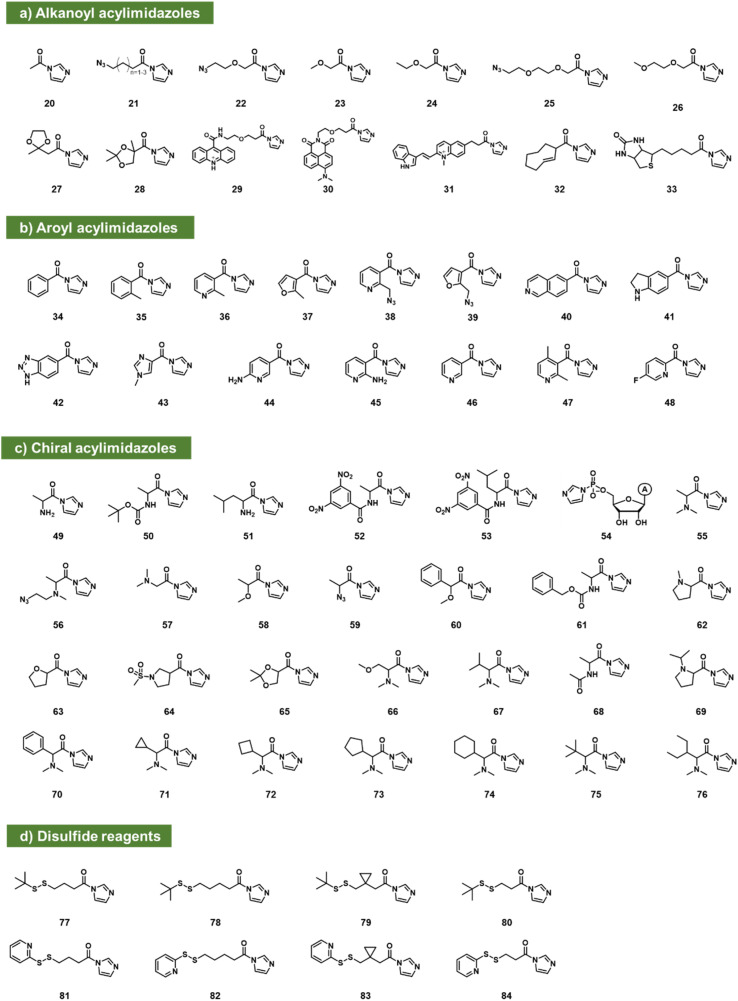
Acylimidazoles reported to react at 2′-OH groups in RNA. (a) Acylimidazole derivatives of alkanoic acids; (b) acylimidazoles derived from aromatic carboxylic acids; (c) chiral acylimidazole species that have the potential to react stereoselectively with RNA (stereocenters not shown explicitly; each species (except 54) exists as a pair of enantiomers, which were tested separately). (d) Acylimidazole reagents with reductively-cleavable disulfide groups.

Acylimidazoles were among the earliest classes of reagents tested for reaction with RNA (20–84; Fig. 3). Acetylimidazole (20) and benzoylimidazole (34) were described several decades ago as acylating agents for RNA;^50^ researchers used radiolabeled reagents to quantify yields of 20–26% per tRNA strand. Acylimidazoles are conveniently prepared in one step from carboxylic acids *via* reaction with carbonyldiimidazole (CDI). Early studies of amino acids converted to acylimidazoles were carried out to test prebiotic chemistry hypotheses of the origins of amino acid-charged tRNAs.^51,52^ Reaction yields of up to ∼10% for alanine reagent (49) and other amino acid species (*e.g.*50–53) were documented with dinucleotides, and a few reactions were performed with homopolymer RNAs. It was later noted that having a free primary amine group on amino acids led to decomposition, likely due to polymerisation.^53^ In related work aimed at elucidating the prebiotic origins of tRNA aminoacylation, adenosine-5′-phosphorylimidazolide (54) was utilized as an aminoacyl acceptor, enabling the transfer of dl-serine to adenosine-5′-(*O*-methyl phosphate) with a preferential transfer of the d-isomer^54^ (Fig. 3c).

Moving beyond prebiotic chemistry, Spitale *et al.* surveyed potential acyl reactants for RNA, and benzoylimidazole 35 gave promising levels of reaction.^7^ Reaction with CDI in anhydrous DMSO created a 1–2 M stock solution of the acylimidazole (along with an equivalent of the byproduct imidazole), and the stock reagent was added to the aqueous RNA solution to yield final concentrations in the 100–400 mM range. To increase solubility, the phenyl group of 35 was replaced with heterocycles pyridine (36; NAI) or furan (37; FAI). The electron-poor pyridine renders NAI more reactive with RNA but gives it a shorter lifespan toward hydrolysis in water (*t*_1/2_ = 33 min). FAI, with its electron-rich pi system, survives longer in water (73 min *t*_1/2_) but gives lower yields in preparative reactions with RNA.^55^ Both reagents are cell permeable and were subsequently used in mapping RNA structures in intact cells.^7,55,56^

An azide-modified form of NAI (38; NAI-N3) has shown utility both for in-cell mapping of the transcriptome^12^ and for high-yield modification of RNAs.^13^ Post-reaction, the azide group can be appended with biotin (*via* strained alkyne cycloaddition) to enrich reacted RNAs from unreacted ones, conferring improved signal over background.^12^ Similar azide-enabled modification for structure mapping was demonstrated with the furoyl species 39 as well.^57^ In tests of the possibility of superstoichiometric reactions, it was subsequently shown that 38 can modify over 80% of 2′-hydroxyls of an RNA strand, documented by MALDI-TOF mass spectrometry.^13^ The high-yield reaction was carried out in pH 7 buffer with 100 mM reagent (2–4 h). Little or no reaction with DNA was observed, demonstrating selectivity for 2′-OH on RNA. Polyacylation of ribozymes and fluorescent RNAs with 38 (also referred to as “cloaking”) was used to temporarily block folding and interactions, and the azide group could serve as a trigger for self-aminolysis of the ester after azide reduction by a reducing phosphine (see below). This enabled its use in chemical caging of RNAs.^13^ The same reagent was subsequently employed to modify CRISPR single guide RNA for control of CRISPR-Cas9 gene editing in two independent laboratories.^58,59^ The compound NAI-N3 (38) was also applied more recently in strategies developed for local preparative installation at sequence-programmed sites in RNAs.^60,61^

The concept of chemical caging of RNA *via* polyacylation of 2′-OH has subsequently received a good deal of attention, frequently using acylimidazole species. Examples (in addition to 38) include a *trans*-cyclooctene reagent (32)^44^ that is reversibly triggered by an amino-tetrazine, and a cyclobutanone adduct (167; see Fig. 6) that is reversed by histamine,^62^ and a biotin-substituted acylimidazole (33) that associates with streptavidin to block RNA activity.^63^

A survey of acylimidazoles reacting with RNA (see 21, 22) showed that multiple such species can react at superstoichiometric levels with RNA in water.^63^ In addition to the original aryl compounds, acylimidazole derivatives of alkyl carboxylates in general have also been found to react well. Park *et al.* reported the use of linear-chain species based on alkanoic acids or similar compounds with ethylene glycol linkages. These were substituted at the terminal position with azide groups for further reaction and conjugation.^44^

Marinus *et al.* reported tests of several aryl acylimidazole species (40–46) and investigated their capability as structure mapping reagents at trace yields.^43^ No data were reported regarding their ability to react at stoichiometric levels, but compound 45 (2AI) was reported as a promising mapping reagent for RNAs in intact cells, with especially good signals in bacteria.^43^ Chen *et al.* recently reported a structurally related fluorine-substituted pyridyl reagent (48) as an NMR reporter for RNA structure.^64^

Xiao *et al.* recently reported a survey of alkyl and aryl acylimidazoles with varying substitution and steric size, investigating their yields and selectivity for reaction among widely varied RNA structures.^65^ Compounds 25–31 and 47 were studied, and a wide range of reactivities was observed. Because both RNA structure and reagent structure affect local reactivity, a sequencing-based study of their reactivity with a library of 43 RNAs was performed. As was seen for previous compounds used in structure mapping, the new compounds showed a preference for loop nucleotides over those in duplex (stem) structure. More sterically bulky compounds (*e.g.*, comparing 36, 46, 47) were shown to yield more localized signals in small loops. Following up on this for practical application, an azide-substituted compound (25) was prepared in one step from commercial precursor, and applied for local reaction at a single nucleotide in an RNA target, achieving preparative-scale fluorescent labeling.^65^

Since RNA is chiral, this offers the possibility of asymmetry in reactions with chiral electrophiles. In this vein, Shioi *et al.* investigated the effects of chirality on high-yield acylation of RNAs by simple acylimidazoles derived from amino acids and alkoxy acids (55–65).^66^ Early surveys showed that enantiomers of acylimidazole derivatives of methoxypropionic acid (58) and *N*,*N*-dimethylalanine (55) reacted with significant (2 : 1–4 : 1) diastereoselectivity. Following up on this, the authors also prepared an azide-substituted variant of the species ((*R*)-56) and employed it in site-localized high-yield labelling of RNA. One surprising observation from the study was the unusually high reactivity of (*R*)-*N*,*N*-dimethylaminoalanine acylimidazole (55), which reacted with RNA to give superstoichiometric yields at unusually low concentrations of 100 μM, 100 to 1000 times lower than standard concentrations of most other reagents, while simply omitting the α-methyl group (compound 49) lowers acylation efficiency by over 10-fold. A more recent study of chiral acylimidazoles with greater steric bulk (66–76), documented up to >99 : 1 diastereoselectivity for compound 76, the most highly stereoselective RNA acylating agent to date.^53^

RNA in general is highly labile to nuclease enzymes, and longer RNA strands are also strongly susceptible to thermal degradation.^67,68^ The mechanism of cleavage of RNAs (both enzymatic and thermal) usually involves attack of the 2′-OH group on the neighbouring phosphodiester. Given this fact, Fang *et al.* tested superstoichiometric acylation at 2′-OH as a strategy to protect RNA from degradation.^17^ However, acylation can also block hybridisation and translation of RNAs,^61^ so for useful application in cells, acyl esters should be reversible. In this light, Fang *et al.* studied a small set of simple acylimidazole compounds (20, 23, 24, 26, 38, 46) for their ability to react in superstoichiometric yields with messenger RNAs (mRNAs) and protect them from thermal and enzymatic digestion. Most acyl groups were found to provide significant protection, and dimethylglycine in particular stabilised RNAs thermally at the elevated temperature of 37 °C for six days. It was subsequently found that small nucleophilic reagents in solution (such as Tris or DABCO) could promote removal of some of the ester adducts from the RNA, and interestingly, evidence suggested that dimethylglycine was spontaneously lost from RNAs in cells, enabling the recovery of protein expression.

More recently, Guo *et al.* designed a series of acylating reagents that introduce disulfide-containing adducts into RNA at superstoichiometric levels. The adducts were designed to be removed by intracellular reducing stimuli (77–84; Fig. 3d). Compound 81 showed enhancement of RNA cellular uptake and distribution without apparent lysosomal entrapment.^69^

### Imidazole carbamates

**Fig. 4:**
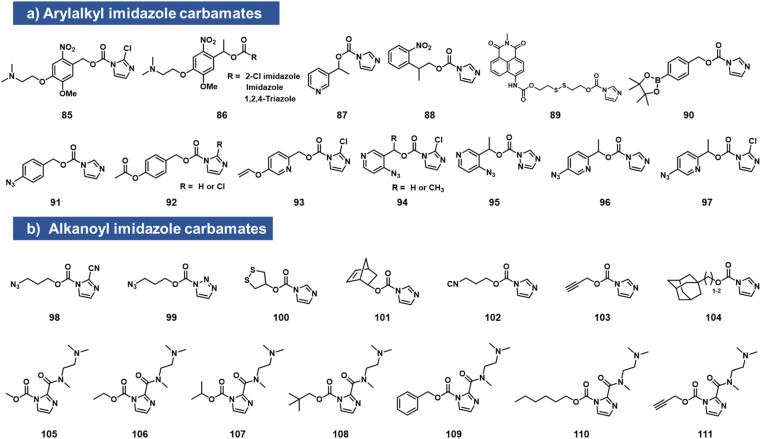
Imidazolecarbamate species reported to react with 2′-OH groups in RNA. To increase reactivity, the imidazole leaving group is often replaced with more active ones, either directly (as in 2-chloroimidazole or 2-carboxamido substituents), or by addition of a nucleophilic catalyst such as DMAP in solution. (a) Compounds with aromatic scaffolds; (b) aliphatic-based reactants.

While imidazole carbamates (Fig. 4) have been known as electrophiles for protein sidechains for some time,^70^ no such studies had been performed with RNA until relatively recently. Velema *et al.* first described the development of an acylating agent derived from an *ortho*-nitrobenzyl alcohol, with the aim of constructing a photocaging agent for 2′-OH groups.^28^ Tests with a simple imidazole carbamate revealed it to be too unreactive, giving little measurable reaction with RNA after 4 h. However, with survey of carbamate leaving groups led to replacement of the imidazole with 2-chloroimidazole (1.5 h *t*_1/2_ in water) increasing its leaving group ability, and the corresponding reagents (85, 86, which also contain a solubilizing dimethylamino group) were able to react with RNA at superstoichiometric levels. The resulting adducts had carbonate linkages that were stable in the dark. This enabled the use of light to switch on biochemical and biological activities of RNA *in vitro* and in live human cells.^28^

Simple imidazolecarbamates (98, 99) can in some contexts provide useful yields with RNA. Reagents (*R*)/(*S*) 87 were shown to react with a short RNA in ∼20% yields.^66^ A different photocleavable reagent (88) was recently reported by Liu *et al.*, using DMAP catalysis to increase reactivity, and applied to control CRISPR-CAS12a activity with UV irradiation.^71^ The CRISPR system has been a test bed for multiple RNA caging approaches using imidazolecarbamate reagents, often assisted in the acylation reaction by DMAP catalysis. Lin *et al.* employed imidazole carbamates and triazole carbamates of photocleavable reagents in the presence of DMAP for modifying CRISPR crRNA, confirming successfully conjugation of RNA *via* PAGE gels.^72^ Similarly, Lei *et al.* used DMAP catalysis to modify CRISPR RNAs with imidazolecarbamates containing disulfide groups (89, 100) that could be reversed by addition of dithiothreitol.^73^ Other chemical caging strategies employed with imidazolecarbamates include a peroxide cleavage design (90),^74^ a norbornene–tetrazine strategy (101),^75^ an isonitrile–tetrazine cycloaddition (102),^76^ mesoporous metal–organic frameworks (103),^77^ an adamantane recognition system (104),^78^ Pd-catalyzed azide reduction (91),^79^ and enzyme-mediated deacylation (92).^80^ Employing the chloroimidazole leaving group, Park *et al.* reported studies of imidazolecarbamate reagents that use quinone methide mechanisms for triggering removal of acyl esters on RNA.^81^ Compounds 93–97 were shown to react with RNA at superstoichiometric levels. The compound adducts on RNA could be reversed with varying rates by addition of water-soluble phosphines. The most successful compound was 97, which could be added to RNA at a level of several adducts per strand, and then could be removed in *ca*. 1 h by addition of TPPMS phosphine (0.5 mM) or TCO-OH (5 mM).

Velema *et al.* described the use of a dialkylamino-substituted imidazole leaving group to activate other imidazole carbamate species.^82^ This elevated the water solubility of otherwise poorly soluble species (105–111), and made it possible to decorate an RNA strand with several hydrophobic groups, which remarkably rendered the RNA soluble in organic solvents. In addition, the same soluble imidazole was used to emplace several methylcarbonate groups on an RNA strand using reagent 105.^82,83^

### Other acyl species

**Fig. 5:**
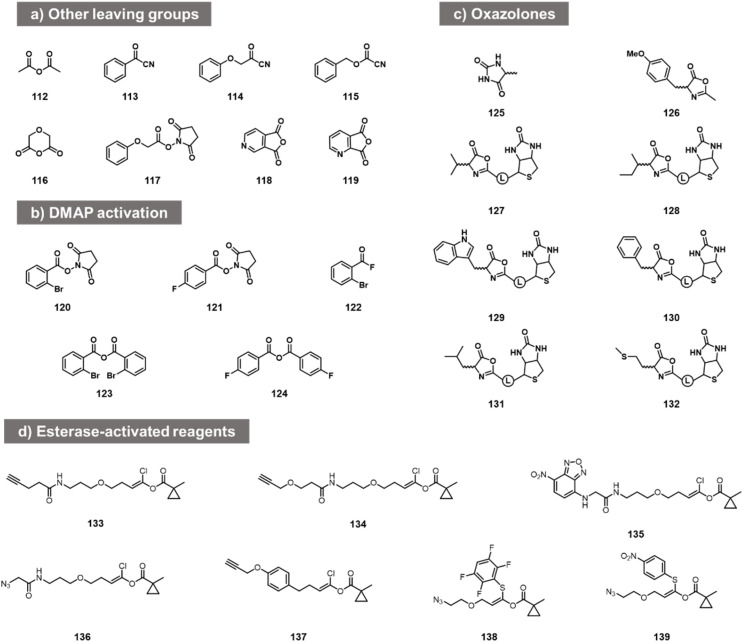
Other acyl reagent types reported to react at RNA 2′-OH. (a) Examples of anhydrides, acyl cyanides, and NHS esters tested for RNA acylation; (b) acylating agents that react with RNA in the presence of added DMAP catalyst; (c) oxazolone electrophiles employed for modification of nucleosides and of RNA 2′-OH; (d) esterase-activated acyl species.

While the above carbonyl electrophiles are the best documented classes of reactants for RNA, many more sp^2^ carbon electrophiles exist, and there are a few reports of tests of their reactivity with RNA. The earliest reports of RNA acylation of transfer RNAs utilized acetic anhydride (112, Fig. 5) in DMF, giving yields of up to 70–92% of one adduct per tRNA.^84,85^ More recently, Spitale *et al.* tested several acyl species including other anhydrides, NHS esters, and acyl cyanides before arriving at the higher-yielding acylimidazoles (113–119).^7,86^ The NHS ester and some of the more hydrophobic anhydrides gave little or no reaction, while more hydrophilic anhydrides gave low levels of apparent acylation. Acyl cyanides were apparently too reactive, giving very short lifespans in water. In addition to this, in patent applications, Goldsborough envisioned the RNA reactions of species such as acyl chlorides, acyl bromides, and anhydrides with RNA, although yields were not given.^87^

More recent studies with some of these acyl species (notably NHS esters 120, 121, acyl fluorides 122 and anhydrides 123, 124) showed that preparative-level yields in reaction with RNA can occur in the presence of the transacylation catalyst DMAP, observing substantial modification with RNA from the HIV-1 R region,^88^ although the quantitative yields were not provided. This catalyst strategy likely serves both to increase reactivity and solubility, and provides enhanced versatility by enabling the use of multiple precursor types.^88^

Other acyl species have also been studied recently. Oxazolones were described as a novel class of activated acyl species for reaction with the 3′- terminus of RNA at 3′- and 2′-OH. Liu *et al.* described reagents (125, 126; Fig. 5c) and documented reaction yields up to 30% with adenosine derivatives.^89^ Similarly, biotinylated oxazolones (127–132) were tested with ribozymes, and stoichiometric reactions were observed in gel shift assays (quantitative yields of acylation were not reported).^90^ Finally, chlorovinyl and related esters 133–139 have been reported by Pani *et al.* recently (Fig. 5d);^91^ these are designed to be activated by intracellular esterases to generate reactive acyl species (*e.g*. acyl chlorides) *in cellulo*. Tests with RNA alone showed reaction and labelling, although stoichiometric yields were not measured.

### Bifunctional electrophiles

**Fig. 6:**
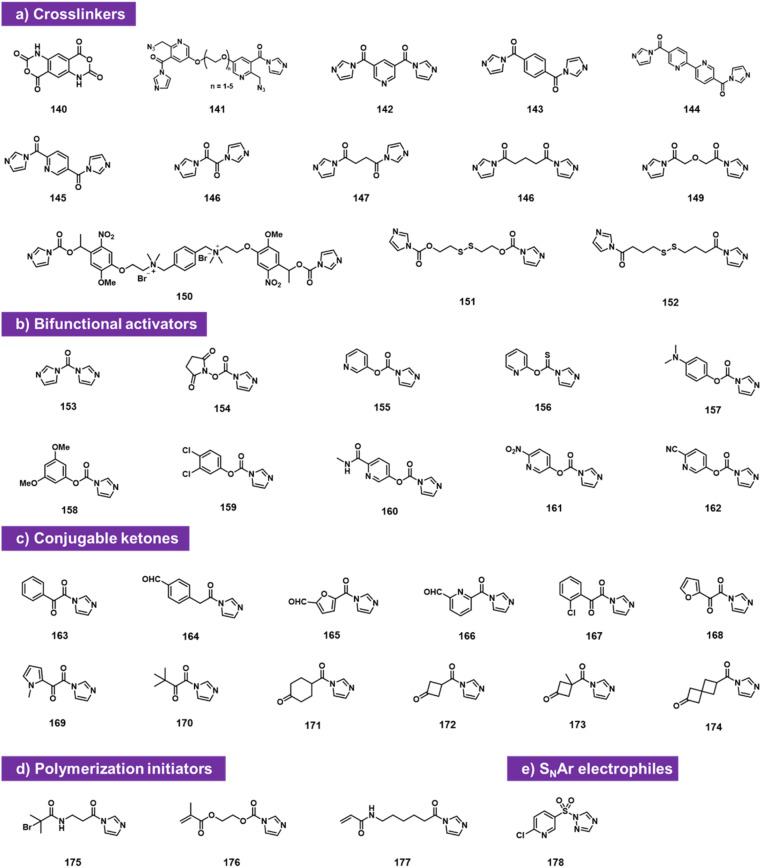
Doubly electrophilic reagents that react with aqueous RNA 2′-OH groups. Reagents are used as (a) RNA–RNA crosslinkers; (b) as activators that install electrophilic carbonyl groups at the 2′-O position of RNA; (c) as reagents for installing reactive ketones; (d) as initiators for polymerisation; and (e) as activating agents for conjugation *via* S_N_Ar chemistry.

Bifunctional electrophiles (Fig. 6) offer the possibility of reaction with RNA and subsequent second reaction with another nucleophile. A recent study reported that carbonyldiimidazole 153 reacts rapidly with both RNA and DNA to provide imidazolecarbonyl adducts in superstoichiometric yields.^92^ The fact that DNA adduct yields were substantial (although below those of RNA) suggests strongly that a substantial fraction of the acylation occurs at exocyclic amine groups of the nucleobases, along with some reaction at 2′-OH in RNA, likely due to the very high reactivity of CDI. To attenuate reactivity, the authors replaced one of the imidazole groups with a series of phenolic leaving groups (154–162). They identified reagent 160 as an RNA-selective reagent for modification at 2′-OH. When incubated at 200 mM with RNA at pH 7.5 buffer supplemented with imidazole, the reagent provided high selectivity for RNA over DNA, resulting in several imidazolecarbonyl adducts. These are transiently stable in water (half-life on the order of 1 h), but offer the strategy of conjugation with nucleophiles (amines and thiols). Reaction with primary amines and thiols proceeded rapidly and in high yields, producing stable carbamate and thiocarbonate linkages and conjugates.

Also falling into this category are bis-electrophiles designed for RNA–RNA crosslinking. Doubly reactive bis-acylimidazoles and bis-isatoic anhydrides have been studied for this purpose, and applied to analysing RNA–RNA contacts and distances. Named probe types include TBIA (140), BINARI (141), DPI (142), SHARC (143–149),^93–96^ and several others (150–152).^72,73^ Also studied as doubly reactive species for RNA are reagents that acylate RNA and then initiate polymerisation,^97,98^ for application in constructing RNA-polymer hybrids. Finally, sulfonyl triazole reagent 178 can sulfonylate RNA and then serve as an S_N_Ar substrate for RNA conjugation with thiols.^99^

A commonly useful electrophile for bioconjugation is the carbonyl group of ketones. Multiple laboratories have reported the installation of ketones into RNA *via* isatoic anhydride (19)^49^ or acylimidazole reactivity (163–174).^62^ The ketones can then be reacted with aminooxy-substituted molecules (examples include biotin or fluorescent labels), resulting in oxime conjugates. Among the more recent ketone species, cyclobutanone compound 172 proved to be both highly reactive to RNA and readily conjugable with hydrazines; it also was selectively removed from RNA with histamine (Fig. 6c).^62^

### Sulfonyl species

**Fig. 7:**
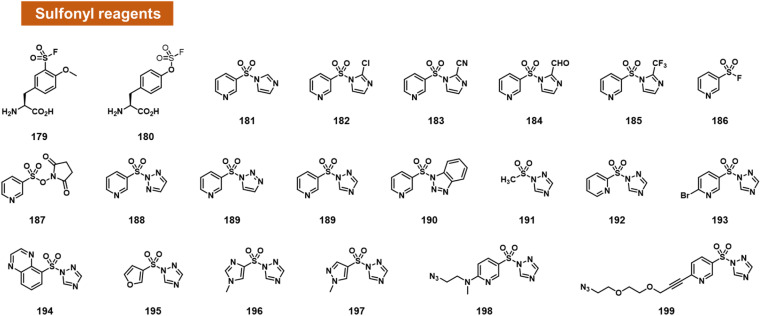
Activated small-molecule sulfonyl reagents that react at RNA 2′-OH groups. Among the leaving groups studied to date, 1,2,4-triazole appears to provide the highest RNA reactivity (*e.g.*189, 199).

Small activated sulfonyl species have come under intense study in the recent decade for aqueous reactions with protein sidechains. For example, sulfonyl fluoride and sulfonyl triazole species have sufficient aqueous stability to survive in water for a substantial length of time, and remain reactive enough to modify hydroxy, amine, and sulphur-containing sidechains in proteins (Fig. 7).^100,101^

Based on this precedent, Sun *et al.* recently reported the engineering of proteins with reactive sulfonyl fluoride and fluorosulfate groups on aromatic amino acids (Fig. 7).^102^ These modified proteins were used to trap interactions with RNA in the cell, by reaction with nearby 2′-OH groups of RNAs bound to the protein. As controls to document the proposed reactions, the authors reacted two fluorosulfonyl substituted amino acids with mononucleotides, and observed up to 16% yield.

Chatterjee *et al.* tested a range of small-molecule sulfonyl species reacting at 2′-OH groups in RNA (Fig. 7).^99^ The authors surveyed different leaving groups for a pyridylsulfonyl (see 181–199), demonstrating highest yields (superstoichiometric in some cases) for sulfonyltriazole species. Little or no reaction was observed with DNA of the same sequence, indicating selectivity for 2′-OH. A test of alkyl sulfonyl species (191) revealed generally little reaction with RNA, possibly because of lower sulfonyl electrophilicity. With extended reaction times (typically 24 h), aryl sulfonyltriazoles could react with RNA with 100% or greater conversions, enabling labelling of RNA by use of an azide-substituted scaffold (199).

Sulfonyltriazoles can be synthesised conveniently from available sulfonyl chlorides in one step. Although slower to react with RNA than, for example, acylimidazoles, one beneficial property of the sulfonyl-1,2,4-triazoles in the study is their stability; they survive standard silica column chromatography and have attractive stability for storage. An example was found to last for *t*_1/2_ = 1.5 hours in water.^99^

### Arylation at 2′-OH *via* S_N_Ar reactivity

**Fig. 8:**
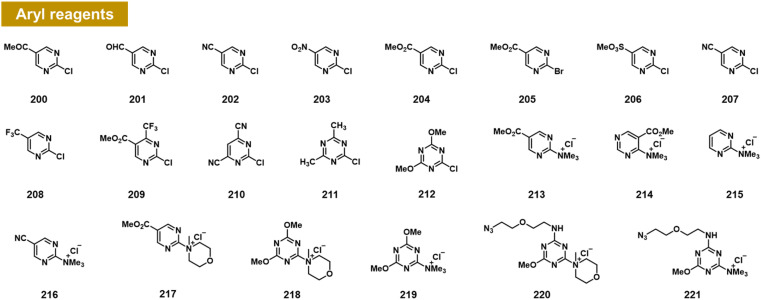
Aryl compounds found to react with RNA 2′-OH groups in preparative yields *via* the S_N_Ar mechanism. Pyrimidines and triazines were found to react in low to moderate yields with chloride leaving groups; however, alkylammonium groups (213–221) greatly increase RNA reactivity and aqueous solubility. Distinct from previous reagents (Fig. 2–7), these produce an ether linkage with the biopolymer.

Chatterjee *et al.* recently documented the surprising finding that RNA 2′-OH groups could react efficiently with aryl electrophiles *via* nucleophilic aromatic substitution (S_N_Ar; see Fig. 8).^103^ They observed moderate but positive reactivity of heterocyclic species such as chloropyrimidines (200–212). However, replacing halogen leaving groups with trialkylamines such as *N*-methylmorpholine (NMM) or trimethylamine (TMM) (see compounds 213–221) resulted in cationic ammonium salts that are much more reactive with RNA, producing >100% conversions with single-stranded RNA within ∼3 h. NMM salts of triazine have been employed previously for activating carboxylic acids for amide coupling,^104^ but were not known to be reactive with alcohols; notably, one RNA-reactive NMM triazine was shown to be stable in pure methanol for a day. Indeed, a NMM triazine salt was shown to be remarkably stable in water (half-life of >10 days), while it reacts with RNA to high conversions in as little as 2–3 hours. Testing the stability of a triazine ether adduct in RNA, the authors found no degradation after a week in buffer at room temperature. An additional feature of the triazine scaffold is its ease of multi-derivatisation; a dichlorotriazine was functionalised in one step with an azide-substituted amine, and then in a second step, reacted with NMM to produce a reagent (221) that can be employed to label RNA in high yields.^103^

### DNA/RNA-catalysed reactions

**Fig. 9:**
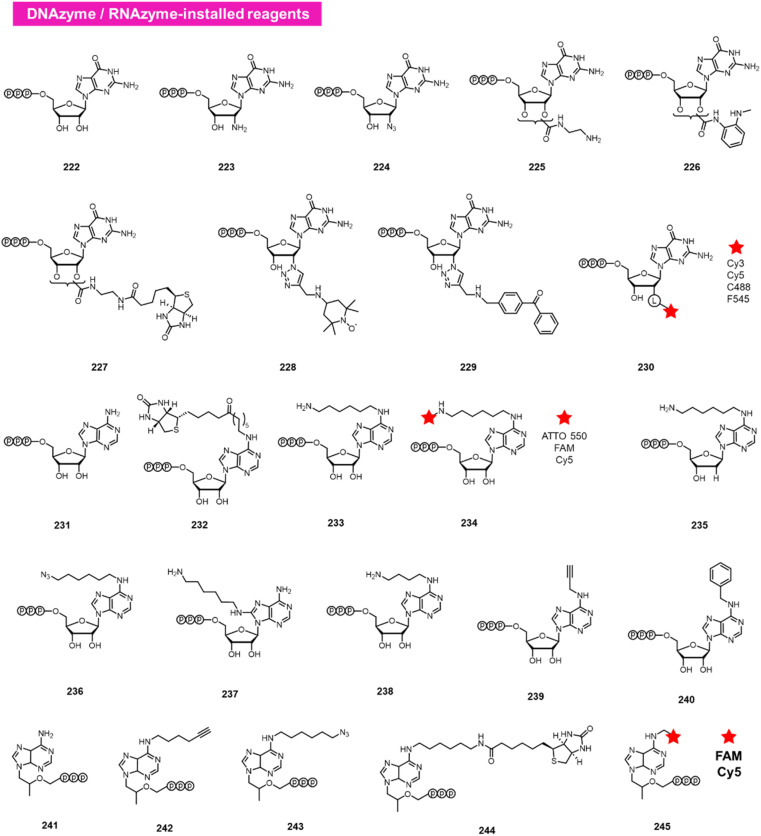
DNAzyme and RNAzyme-directed reactions have been employed to modify RNA 2′-OH. Reagents 222–245 are conjugated to adenosines in RNA with loss of pyrophosphate leaving group.

A different approach to functionalising the 2′-OH group of RNA in water has been developed by Höbartner, who has made use of designer catalytic DNAs (DNAzymes) and RNAs (ribozymes) to transfer nucleotide analogues to the 2′-OH position of RNAs. Early work in the Silverman lab reported the discovery of the 10DM24 DNAzyme, which was shown to transfer nucleotides to the 2′-OH of internal adenosines in RNA strand.^105^ Büttner *et al.* subsequently made use of this DNAzyme, varying the sequence of the “arms” on either side of the active site, to direct the labelling of RNAs *via* GTP analogues (222–230; Fig. 9).^106^ Yields of >70% were reported, and a number of labelled GTP analogues (with labels at the 2′-O position) are commercially available for application. The same lab subsequently discovered a ribozyme that catalyses similar transfers of a different nucleotide, ATP, to adenosines in RNAs (231–240).^107^ In this case, labels are incorporated at the N6 position of ATP. More recently, Maghami *et al.* developed a ribozyme that transfers tenofovir-triphosphate analogues to RNA, which enables dual orthogonal labeling of two sites in RNAs (241–245).^108^ Taken together, the methods offer the favourable ability to direct modification to RNA in a sequence-directed way, with the only apparent limitation of adenosine as the specific labelled site.

### Functional groups and conversions

**Fig. 10:**
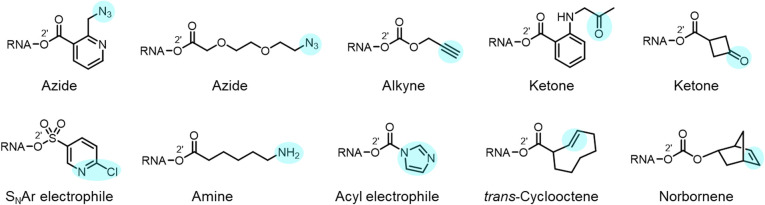
Examples of diverse functional groups that have been incorporated into RNA *via* reaction at 2′-OH groups. In RNA, these functional groups (highlighted in colour) enable further modification *via* cycloaddition and nucleophilic addition chemistries.

Reactions carried out at 2′-OH not only serve to block or alter RNA interactions, but also can be employed to introduce functional groups into RNA, incorporating new reactivity and properties into the biopolymer (Fig. 1b and 10). As mentioned above, adducts with azide groups placed strategically near an acyl linkage to RNA can be reduced with phosphines, resulting in cyclisation and removal of the adduct.^13,44^ However, placement more remote from the acyl group enables azides to be reduced to produce amines as stable groups in RNA.^44^ An example of this is acylimidazole 21 (*n* = 3), which can be used to emplace a primary amine into RNA. Of course, azide groups can also be reacted with alkynes, and this strategy has been employed to conjugate fluorescent labels or biotin groups into RNA *via* azide or alkyne-substituted reagents.^12,60^ The Velema and Zhou laboratories have applied carbamates with alkyne groups to RNA,^77,82^ and Pani has also leveraged esterase-triggered reagents including an alkyne.^91^

Other functional groups have also been introduced into RNA at 2′-OH *via* acylation or sulfonylation chemistry. As mentioned above, bis-electrophiles (140–152) can react with RNA, introducing a second active acyl that can trap other nearby RNAs in a crosslinking strategy.^93–96^ Imidazole carbamate species have been used to introduce photocaging groups into RNA.^28,71^ Ketones can be introduced *via* isatoic anhydride or acylimidazole reagents.^49,62^ As mentioned above, a doubly reactive sulfonyltriazole reagent (178) has been employed to introduce a 2-chloropyridyl group into RNA, which undergoes subsequent high-yield S_N_Ar chemistry with thiols (Fig. 10).^103^

### General principles for design of reagents

While studies of reactions at 2′-OH in RNA are still in their early stages as compared with the large extant body of work on protein derivatisation, the data to date allow an initial assessment of some of the relevant properties that determine the most effective designs of reagents.

#### Electrophilicity and aqueous lifetime

**Fig. 11:**
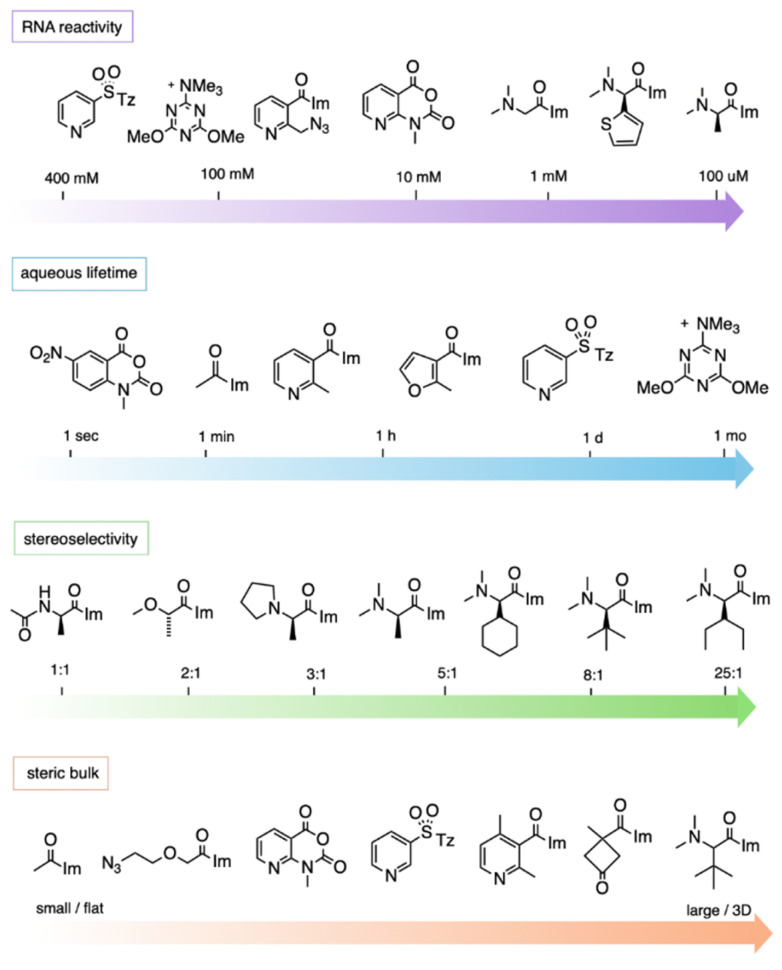
General trends of reagent structures and properties for reaction at 2′-OH groups in RNA, showing representative examples. “RNA reactivity” (top row) shows common concentrations required to yield >1 adduct per RNA strand with example reagents shown; the most highly RNA-reactive reagents can polyacylate RNA at 100 μM reagent. “Aqueous lifetime” illustrates the wide range of reported half-lives of electrophilic 2′-OH-reactive species in water. “Stereoselectivity” depicts diastereoselectivity in reactions of the enantiomers of chiral compounds with single-stranded RNA (favoured enantiomer shown). Stereoselectivity can be higher in folded RNAs. “Steric bulk” shows size of substituents near the reactive electrophilic atom (C or S). “Im”: imidazole; “Tz”: 1,2,4-triazole.

The design of electrophiles for RNA must strike a balance of reactivity (Fig. 11). Elevated reactivity may enhance rates of reaction with the biopolymer, but working against this is the reactivity with water itself. Indeed, some of the most strongly reactive acyl species have very short lifetimes; for example, 1M7 ((2), a nitroisatoic anhydride) has a half-life in water of a few seconds, and reacts in very low yields with RNA.^44^ Conversely, some imidazole carbamates react poorly both with water and with RNA,^19,28^ while other imidazole carbamates show better RNA reactivity, especially in the presence of DMAP catalysis.^71,74–79^ For compounds that do have effective reactivity in preparative yields with RNA, the sulfonyltriazoles such as 181–199 exist at the low end of the reactivity spectrum. They can give stoichiometrically high yields with RNA after 24 h, and have an aqueous half-life on the order of 1–2 h.^99^ More reactive species such as acylimidazoles 38, 55, 71 can acylate most of the hydroxyls on an RNA strand in 1–2 h, and typically exhibit half-lives of *ca*. 5–60 min.^13,72,73^

It should be noted that the measurement of aqueous half-lives for electrophiles is illuminating but not entirely predictive of RNA reactivity. For example, (*R*)-dimethylalanine acylimidazole reagent 55 has a half-life only a few minutes shorter than that of NAI-N3 (another acylimidazole), but the former reacts with RNA in superstoichiometric levels at concentrations 100-fold lower than the latter.^66^ More dramatically, recent arylation reagents have demonstrated greatly extended aqueous lifetimes; for example, compound 218 has a *t*_1/2_ of 10.7 days in D_2_O, while it reacts at high yields with RNA in 2–3 hours.^103^ Given the large disparity of water *vs.* RNA reactivity of this latter compound, these arylating agents are the most RNA-selective reagents yet identified, with a reported ∼10^8^-fold kinetic preference for 2′-OH. The results demonstrate that it is possible to increase selectivity for RNA 2′-OH over water, but it is currently difficult to predict which species will exhibit the highest selectivities.

#### Adduct stability and reversibility

The addition of a carbon acyl group to a 2′-OH group in RNA results in an ester linkage of varying stability in aqueous solution. For many applications, it is desirable that the ester is stable enough to remain intact during whatever experiments or analysis are planned for the modified RNA. For structure mapping, that may be only an hour or two, whereas for fluorescence labelling the conjugated RNA may be used in experiments over weeks. While general stability data are not yet widely available for the different acyl adducts reported for RNA, some early data have been reported. Esters of electron-poor heterocycles are generally less stable than electron-rich ones; for example, NAI adducts on RNA are reported to be less stable than FAI adducts;^55^ although NAI adducts are known to be stable enough for RNA structure analysis, this may be an issue for long-term storage with RNA. It seems likely that similarly electron-poor isatoic anhydride adducts with RNA (*e.g*. 2, 3) may also have limited stability, although we are unaware of data on that issue. A third example is adducts of cationic reagent dimethylglycine on mRNAs; the data show that the adduct spontaneously hydrolyses from RNA over a period of a few hours depending on the buffer, whereas a less electrophilic (neutrally charged) alkoxy reagent forms a much more stable adduct.^17^ Finally, arenesulfonyl adducts^99^ are likely to be considerably more stable than arene acyl adducts, as sulfonate esters require harsher conditions to hydrolyse. Although less data are available, other adducts such as carbamates, carbonates, and aryl ether adducts are likely to be highly stable.^81,92,103^

In some applications, reversibility of the adduct on RNA is desirable. For example, in caging RNA, a triggering mechanism for easy reversibility is necessary. This has been achieved by incorporating light-cleavable groups as mentioned above,^28,71^ or chemically cleavable groups incorporating reducible azide groups.^13,58,79,81^ A second application is the use of acyl groups for protection of RNA from hydrolysis; the presence of the groups inhibits RNA degradation, but eventually the groups may need to be removed so as not to interfere with biological activity. With this in mind, Fang *et al.* described the use of non-basic nucleophiles to promote the loss of acyl esters, and also documented loss of the groups (such as the aforementioned dimethylglycine, 57) inside cells, restoring translation of the RNA.^17^ Multiple labs have successfully designed groups (38) that can be removed in cells by phosphines added to the growth medium, enabling control of RNA-mediated activities.^58,59^ Finally, Guo *et al.* recently employed disulfide acyl adducts (81, 82) that are reversed by reduction both outside and inside cells.^69^

#### Steric effects

RNA 2′-OH groups, as secondary alcohols with phosphate groups adjacent in a large biopolymer, present a relatively sterically occluded environment for approach of reagents. In addition, RNA folded structure can present even greater barriers, as is seen in helices where these groups are even more protected than in single-stranded RNA.^39,109^ Thus it is not surprising that steric bulk in acylating agents might play substantial roles in reactivity and selectivity. Although few systematic studies yet exist on this topic, some comparisons have been made in this regard (Fig. 11). For example, increasing steric bulk at the α-position of amino acid derivatives (*e.g*. 55, 67, 75) slowed RNA reactions, but increased stereoselectivity and ester adduct stability.^53^ Similarly, three pyridyl acylimidazoles (36, 46, 47) with varied steric bulk were compared for selectivity among loop positions in an RNA library.^65^ The results showed that increasing bulk tended to result in greater selectivity among loop positions along the same loop. It should be noted that such selectivity can either be desirable or undesirable depending on the application: for structure mapping, ideal reagents might show similar levels of reactivity along a loop. However, for local conjugation reactions, one might seek reagents that react at only a single position in an induced loop.^65^

#### Stereochemistry

As described above, studies have shown that reagent chirality can have large effects on reaction rates and yields with RNA 2′-OH groups (Fig. 11).^53,66^ It has been shown that both the chirality of the reagent and the local structure of the RNA have important influences on reaction. One useful outcome of chirality is the use of an (*S*) alkoxy-acid compound in mapping RNA structure with higher signal over background (“loop to stem ratio”) than common mapping reagents.^66^ Overall, for future compound design, if asymmetric reagents are considered, one should take into account the existing data on reactive loops, and some further experimentation may well be needed with new loop sequences to determine best compatibility between reagent asymmetry and RNA macrochirality near the intended reaction site.

#### Structure and accessibility of RNA

Mechanistic studies of 2′-acylation of simple mononucleotides have shown that they are 3–7-fold more reactive than dinucleotides, likely due to steric effects of stacking of adjacent nucleobases, which hinders access to the 2′-OH group between them.^21^ In single-stranded (non-folded) RNAs, it has been shown that some reagents are much more reactive to polyU than polyA,^109^ which has been attributed to the fact that adenine base stacks considerably more strongly than uracil.

Unpaired nucleotides in RNA are generally several-fold (generally 2–8-fold) more reactive than those in duplex (paired) structure. Loops are highly variable in their reactivity. Early studies of ribosomal RNAs by Weeks showed that not all unpaired nucleotides react equally with acylating agents. A study of NAIM revealed that there were “hyper-reactive” positions in certain loops of this context.^22^ In the study, this was attributed to possible general base catalysis by nearby nitrogens on bases. A more recent study by Xiao *et al.* carried out a quantitative survey of the effects of different loop and unpaired contexts on reactivity to the common reagents 1M7 and NAI-N3.^109^ A library of RNAs with systematically varied loop sizes and types, along with three single-stranded RNAs, was profiled at every position for reactivity, with data obtained by deep sequencing. The results showed, surprisingly, that very small loops were the most reactive in the library, while increasing loop sizes tended to decrease reactivity. This runs counter to the notion that increased RNA flexibility might favour reaction. Certain loop types displayed the highest reactivity; for example, 1–3 nt bulge loops and small asymmetric internal loops were among the most reactive motifs in the library, and proved to be 50-fold to 4000-fold more reactive than purely single-stranded RNA of the same sequence, likely by exposing specific 2′-OH groups in relatively rigid way.

From a practical standpoint, highly selective RNA loop reactivity can be important as a strategy for reacting at specific 2′-OH groups in a larger RNA. Xiao *et al.* have used complementary DNAs to induce small reactive loops at designed sites in RNAs, enabling sequence-specific modification (*e.g.* for fluorescence labelling without interfering with biological activity).^61^

## Conclusions and future prospects

The early research in this field has pointed to high promise of applications of 2′-OH reactivity in RNA, and the chemical and structural diversity of reagents that undergo this reaction has rapidly increased in the last few years. Reagents are useful for mapping RNA structure *in vitro* and in intact cells; for labelling and functionalising RNA; for controlling RNA function; and for protecting RNA against hydrolytic degradation. To date, the most well-studied classes of reagents for trace-level reactions are the isatoic anhydrides and the acylimidazoles. For reagents that yield stoichiometric and superstoichiometric levels of reaction, a few isatoic anhydrides are known, and numerous acylimidazole species, from both alkyl and aryl scaffolds, have shown much utility and structural diversity.

In addition to those two classes of reactive species, imidazolecarbamates, although often less reactive than acylimidazoles, have shown increasing utility in high-yield reactions with RNA, especially with the help of better imidazole leaving groups and nucleophilic catalysis. Aryl sulfonyltriazoles and other sulfonyl species can react, although research on this class of reactants remains in its early stages. Also at early stages is the study of chiral compounds, and the study of S_N_Ar arylation. More work on these classes of reagents is needed to better understand optimal reagent designs and their sensitivity to varied RNA folded sequences and structures. In addition, other active acyl, aryl, and sulfonyl species beyond those tested with RNA to date are known in the literature, and studies of many of those species with RNA are merited. For new reagent species, issues of ease of preparation, solubility and half-life in water, RNA reactivity and RNA structure selectivity should all be kept in mind.

Given the growing versatility of 2′-OH-reactive reagents, it seems certain that further development of specialised reagents is coming in the near future, and new applications are likely to be reported as well. One interesting challenge is the development of new strategies for reacting at specific 2′-OH groups in an RNA strand (as opposed to random reaction); although useful inroads have been made,^44,102^ further research toward this goal seems likely.

## Data availability

No primary research results, software or code have been included and no new data were generated or analysed as part of this review.

## Author contributions

R. S. and E. T. K. conceived and wrote the manuscript.

## Conflicts of interest

Both authors are inventors on patent applications involving 2′-modification of RNA.
